# Congenital Tracheoesophageal Fistula in Very Low Birth Weight Preterm Neonate with an Oligohydramnios as a Rare Presentation: A Case Report

**DOI:** 10.31729/jnma.5207

**Published:** 2020-12-31

**Authors:** Anu Maharjan, Amit Kumar Sharma, Prerana Kansakar, Samyukta K.C, Yogendra Singh

**Affiliations:** 1 Department of Surgery, Patan Academy of Health Sciences, Lagankhel, Lalitpur, Nepal; 2 Laligurans Hospital, Talchhikhel-14, Lalitpur, Nepal; 3 Department of Pediatrics, Patan Academy of Health Sciences, Lagankhel, Lalitpur, Nepal

**Keywords:** *Congenital tracheoesophageal fistula*, *oligohydramnios*, *preterm baby*, *very low birth weight*, *case report*

## Abstract

Tracheoesophageal fistula is a challenging anomaly with a rare prevalence with symptoms mainly respiratory, sometimes digestive. We present a rare case of oesophageal atresia with distal Tracheoesophageal fistula in a female child whose mother presented with severe oligohydramnios on ultrasonography with intrauterine growth retardation before caesarean section. After the birth of preterm and very low birth weight neonate, we initially diagnosed as respiratory distress syndrome with club foot. However, we diagnosed oesophageal atresia with distal Tracheoesophageal fistula on 2^nd^ day as nasogastric tube insertion was failed beyond 10cm and confirmed by X-ray with rubber catheter. Right thoracotomy with ligation of the fistula with end to end anastomosis was performed successfully without complications. Breastfeeding initiated and the child discharged after she started gaining weight. Early post-operation complication (anastomotic stricture) was noticed after 2 weeks; however, corrected with endoscopic balloon dilatation. Currently, the child is healthy weighing 10kgs at 18 months age.

## INTRODUCTION

Tracheoesophageal fistula (TEF) is a birth defect with an abnormal communication between the trachea and the oesophagus.^1^ This rare condition affects one child in 2500-4500 live births.^2^ Type C is the most common among 5 types of TEF, depending upon the location of atresia and associated fistula.^3^ The hallmarks of TEF is copious salivation associated with choking, coughing, vomiting, and cyanosis with the onset of feeding.^1,2^

We present a case of oesophageal atresia (EA) with distal TEF in a female infant delivered at 31 weeks of gestation with very low birth weight whose pre-delivery USG showed severe oligohydramnios with IUGR.

## CASE REPORT

**Figure 1:**
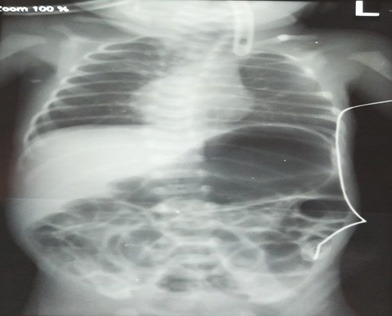
X-ray showing red rubber tube not passing beyond 10 cm and gastric distension indicating oesophageal atresia with tracheoesophageal fistula.

A 24 years old primigravida who had previous normal antenatal (ANC) visit along with USG at 17 weeks showed single live fetus at 16 weeks of gestation (WOG) without any gross fetal anomalies at Darchula district hospital, presented with the chief complaints of pain abdomen and per vaginal leaking for 1 day at 31 weeks of gestation in the same hospital and was referred initially to nearby tertiary care Seti Zonal Hospital. Further, she was referred to our centre (Patan Academy of Health Sciences). Repeat USG was done at 31 weeks which revealed severe oligohydramnios with amniotic fluid index (AFI) 1.5cm and intrauterine growth retardation (IUGR) with the expected fetal weight of 1365 grams only. Emergency lower segment caesarean section was performed with prior administration of 2 doses of dexamethasone (12mg) and prophylactic antibiotics to the mother. A female baby was born with a birth weight of 1.2 kilograms and was immediately transferred to the neonatal intensive care unit (NICU) due to respiratory distress. There was no gross congenital anomaly (no murmur) noted except the club foot. She was managed conservatively with intravenous fluids, antibiotics and non-invasive ventilation (Continuous positive airway pressure (CPAP) at 6 centimetre water) with an initial diagnosis of respiratory distress syndrome with a pre-term baby with very low birth weight. Further, NG tube insertion was tried but failed to pass beyond 10 cm for feeding purpose. Therefore, a red rubber catheter was inserted and a chest X-ray (Figure 1)

**Figure 2:**
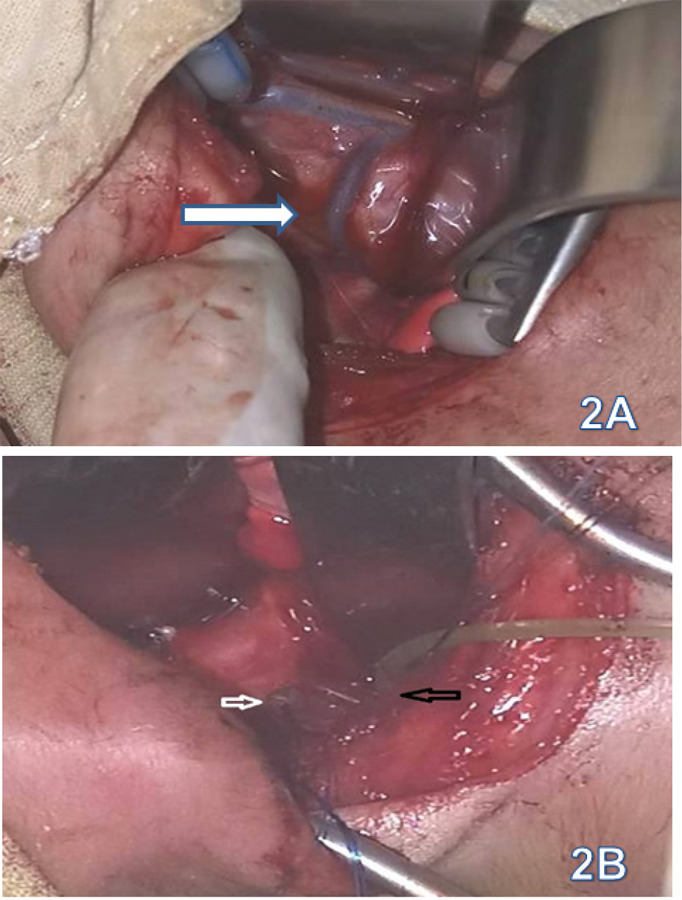

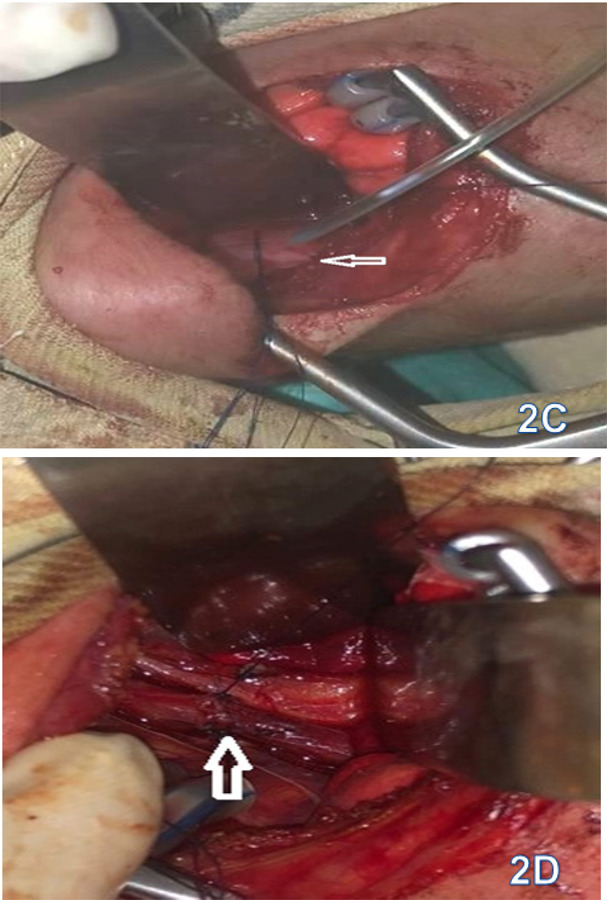
2A- White arrow showing Azygos vein before ligation, 2B- White arrow showing ligated fistula and black arrow shows distal esophagus, 2C- Arrow shows proximal esophagus with feeding tube, 2D- Arrow showing end to end esophageal anastomosis.

was performed which demonstrated EA with TEF. Eventually, surgical correction was planned with all the pre-operative evaluation done to rule out other associated anomalies and complications. Right thoracotomy with ligation of the fistula with end to end anastomosis was performed without any perioperative complications (Figure 2A, 2B, 2C, 2D).

**Figure 3:**
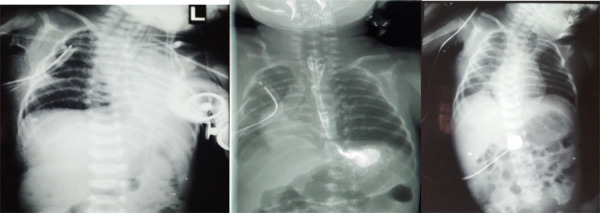
(A) Post-operative X-ray showing atelectasis of left lung and chest tube in right lung. (B) Fluoroscopic evaluation demonstrating patent oesophagus without mediastinal spillage of dye and bilateral normal expansion of lungs field. (C) X-ray demonstrating proximal oesophageal dilatation revealing post-surgical oesophageal stricture.

Additionally, a prophylactic chest tube was kept in situ and the NG tube was fixed. Furthermore, post-operatively continuous monitoring of vitals, haemoglobin, electrolytes, nasogastric and chest tube care, antibiotics coverage (Piperacillin-tazobactam + Metronidazole), and maintaining neutral neck position were done. Repeat chest X-ray on the first post-operative day (POD) showed atelectasis of the left lung and chest tube in situ on right (Figure 3A). NG feeding was started on the second post-operative day and endotracheal tube extubated on day three. Initially kept on RAM cannula and then on continuous positive airway pressure (CPAP). There was no collection in the chest drain. The upper gastrointestinal study was carried out on the 7th POD which was inconclusive with right-sided pneumonia. Antibiotics upgraded to Meropenem and Colistin. Fluoroscopic evaluations on 9th POD demonstrated patent oesophageal lumen with dye in the stomach without tracheal and mediastineal spillage and normal expansion of the bilateral lungs field (Figure 3B). Therefore, oral feeding (as tolerated) started on the same day followed by oro-gastric feeding; however, sucking reflex was absent. The chest tube was removed on the 10th POD. Complete breastfeeding commenced on 21st day and transferred to the children ward and subsequently discharged at weight 1900gm. The patient came on emergency after 2 weeks with complaints of choking, chest X-ray done which revealed oesophageal stricture which is one of the early post-surgical complications (Figure 3C). The oesophageal stricture was endoscopically managed with balloon dilatation. She is under regular follow-up and since has been uneventful. Her current weight is 10 kg at 18 months of age.

## DISCUSSION

Tracheo-oesophageal fistula is an abnormal communication between the trachea and the oesophagus.^1,4^ A rare clinical condition but a challenging congenital anomaly which was first reported in 1697 by Thomas Gibson, has always made managing this condition to be very much strenuous.^2,4^ The abnormality is itself a huge challenge, as a rule, comes in association with one other abnormality (VACTREL: vertebral defects, anorectal malformation, cardiac defects, TEF, renal anomalies, and limb abnormalities).^1^ These abnormalities can be explained as in Table 1. The TEF classification is determined by the location of the oesophageal atresia and the presence of any associated fistula to the trachea.^2^ Five different variants have been clinically described, the first described by Vogt in 1929, and modified by Gross in 1953. Thus, two classifications are used today which includes: (i) EA with distal TEF (85%, Vogt IIIb, Gross C), (ii) Isolated EA without TEF (8%, Vogt II, Gross A), (iii) TEF without atresia or H-type TEF (4%, Gross E), (iv) EA with proximal TEF (3%, Vogt III, Gross B), and (v) EA with proximal and distal TEF (< 1%, Vogt IIIa, Gross).^2,4^

TEF presents in a new-born by copious salivation associated with choking, coughing, vomiting, and cyanosis during the onset of feeding.^1^ The presentation may vary depending upon the presence or absence of EA. New-borns with EA present immediately after birth with excessive secretions that cause drooling, choking, respiratory distress, and feeding inability. Gastric distension is a common complication of a fistula between the trachea and the distal oesophagus. In EA subsequent inability to swallow typically cause polyhydramnios in utero, a common finding on antenatal USG scanning.^1,2^ However, in our case, the mother presented with oligohydramnios which led our doubts overshadowed towards correct diagnosis and hence, following the scan done at 31st weeks of gestation a diagnosis of oligohydramnios with intrauterine growth retardation was made. A very low birth weight infant with the birth weight of 1.2 kgs delivered via a caesarean section without gross anomaly except for club foot. Additionally, there was no excessive secretions. This contrasts with the typical presentation of esophageal atresia. One reason behind the cause of severe oligohydramnios in our case after reviewing the history again could be per vaginal leaking of amniotic fluid. Prenatal diagnosis with USG scans can be helpful when a scan in a mother with polyhydramnios shows the absence of a stomach or a small stomach.^1^ Routine USG scans done between 16-20 weeks of pregnancy can aid the diagnosis but the findings are not specific, are subjective and occasionally transient. This might be due to the reason that polyhydraminos is only diagnostic if TEF is associated with EA. MRI can be complementary to USG as it can be highly predictive although questions have been raised over the feasibility and cost.^1,2^ After birth, if EA is suspected, a radiopaque 8 French (in preterm infants) or 10 French (in term infants) NG or feeding tube should be passed. In patients with atresia, the NG tube coils at 10 to 12 cm.^5^ After passing the tube, chest radiographs (postero-anterior and lateral views) including the entire abdomen should be obtained to confirm the position of the tube.^1,2,4^ In patients with EA, the air in the stomach confirms the presence of a fistula. In our case, as she was delivered pre-term and had very low birth weight, she had a higher risk of developing respiratory distress and so was admitted in NICU (Neo-Natal Intensive Care Unit) and started on intravenous fluids, antibiotics and non-invasive ventilation with a diagnosis of acute respiratory distress syndrome. This misled us to establish the correct diagnosis. However, when a NG tube was inserted in the NICU for the purpose of feeding, the tube could not be inserted; additionally, continuous frothy secretion was noticed only after that point. This gave a clue to us to suspect TEF and hence, a red rubber tube was inserted and a chest x-ray was performed.

Following multiple innovative ways aimed at the correction, it was 1941 when a successful surgical correction was performed by Cameron Haight.^2^ Earlier to this many attempts were made and many surgical procedures tried which failed and this proves how difficult the procedure can be.^4^ The survival rate of such cases which was once hopeless has improved and reached 100%.^5^ For successful management care and precision has to be given since the time of diagnosis and needs special pre-operative, intra-operative and post-operative care and management.^5^ An open surgery which includes thoracotomy with end to end anastomosis of the esophagus with correction of fistula has been reducing the mortality rate of the patients diagnosed with this challenging condition.^2^ Currently various endoscopic and other surgical techniques are being tried in various hospitals with some promising outcomes.^2,6^ In cases of neonates with very low birth weight and extremely low birth weight primary repair of EA and closure of a TEF seems to be a better option amongst the open surgical procedure and has proven to enhance the better outcome than the staged repair;^7^ however controversies exist here as well.^8^ In our case, we performed right thoracotomy with ligation of the fistula with end to end anastomosis without any peri-operative complications. Many surgeons prefer stage repair in VLBW neonate over primary repair; however, here we performed primary repair as patient was from remote region with low socio-economic condition leading to refusal for stage repair or follow ups.

Complications are mostly post-operative and include anastomotic leak (10-20%), anastomotic strictures (30-40%) as in our case which was managed with balloon dilatation, recurrent TEF (5-14%), tracheomalacia (10%), gastroesophageal reflux (40%), and respiratory infections.^1,2,4^ The prognosis may be influenced by the presence of major anomalies such as cardiac malformation, renal deformity, external risk factorssepsis, respiratory failure; it is also influenced by the neonatal caregivers.^9^ Undoubtedly, any efforts to reduce the incidence of the external risk factors may decrease the mortality rate.

The early diagnosis of the case, within 2 days of delivery despite normal antenatal check-up, and the most successfully performed surgery, good post-operative NICU care without any intra or post-surgical complication, are the prime strength of our study. Very low birth weight and prematurity were some of the worth considering factors in the case of our patient as both of them have been taken as factors that adversely affect the outcome;^9^ however, we were lucky enough and our patient proved herself a survivor. Spitz classification deals with classification and survival based on weight.^1,2^ This classification states that the survival rate of infants below 1.5 kgs of birth weight without any major cardiac anomaly to be between 59-82%.^4^ Despite our strengths, we were unable to diagnose this anomaly during pre-delivery (LSCS) USG scanning and the child, unfortunately, developed an early complication as oesophageal stricture; however, oesophageal stricture was successfully managed endoscopically with balloon dilatation.

Although TEF is uncommon in newborn babies, it is wise to USG screening during the antenatal check-up and if doubtful, MRI. Sometimes, they may present with an atypical presentation like in our case with oligohydramnios. Early diagnosis and good care to prevent external risk factors and ultimately surgical correction provide a good prognosis unless the absence of major organ anomalies.
